# Can Yoga Boost Access to the Bodily and Emotional Self? Changes in Heart Rate Variability and in Affective Evaluation Before, During and After a Single Session of Yoga Exercise With and Without Instructions of Controlled Breathing and Mindful Body Awareness in Young Healthy Women

**DOI:** 10.3389/fpsyg.2021.731645

**Published:** 2021-12-03

**Authors:** Cornelia Herbert

**Affiliations:** Department of Emotion and Motivation Psychology, Institute of Psychology and Education, Ulm University, Ulm, Germany

**Keywords:** yoga, heart rate variability, physical activity, emotion, body awareness, self-referential processing, mental health, well-being

## Abstract

Exercise is indispensable for a healthy lifestyle. Yoga exercise can have positive effects on well-being and on cardiac autonomic activity making it an ideal intervention for improving mind-body interactions and resilience to physical and mental stressors. Emotions trigger especially strong bodily and affective-cognitive responses because of their social relevance for the self and their biological relevance of mobilizing the organism for action. This study investigates whether changes in emotion processing related to self-other referential processing and changes in cardiac autonomic activity, reflected by heart rate variability (HRV), occur immediately after already a single session of yoga exercise when yoga postures are practiced with or without breathing- and mindful body awareness instructions. Women, all university students (*N* = 34, final sample: *n* = 30, *n* = 25 naïve to yoga practice) were randomly assigned to two experimental groups who performed the same yoga exercises with or without controlled breathing and mindfulness instructions. Emotional, self-other referential processing, awareness of bodily signals and HRV indicators were investigated before and after the exercise using standardized experimental tasks, standardized questionnaires, and mobile recording devices. Exercising for 30 minutes changed cardiac activity significantly. HRV measures showed adaptability of cardiac activity during the exercise as well as during the affective task post- to pre-exercise. Exercising with breathing instructions and mindful body awareness had no superior effects on cardiac, particularly parasympathetic activity, compared to practicing the same movements without such explicit instructions. Self-referential processing did not change; however, participants were faster and more accurate in their affective judgments of emotional stimuli [regardless of their reference (self/other)], and showed better awareness of bodily signals after compared to before the exercise session. The results support immediate, adaptive effects of yoga exercise on cardiac and affective-cognitive processing in an all-female healthy sample. Therefore, yoga exercise could be recommended as a physical activity for boosting cardiac and emotional resilience in this target group.

## Introduction

### Yoga, Regular Physical Activity, Exercise, and Health

Physical activity (PA) and regular exercise (RE) are indispensable for a positive, health-promoting lifestyle. PA and RE are among the top ten activities recommended by the World Health Organization (WHO, 2010) for health promotion and disease prevention of non-communicable (NCD) and life-style related diseases (LSRD). PA and RE improve overall well-being (e.g., Hosker et al., 2019), buffer stress, anxiety, and depressive symptoms (e.g., Ströhle, 2009; Carek et al., 2011). The physical (i.e., physiological, neurobiological, and neural) and psychological mechanisms by which PA and RE can achieve such positive health effects have been intensively investigated in the past. Evidence converges that there is no single super factor but that effects of PA and RE on health are multifactorial. Physical and psychological factors and their dynamic interaction play an important role.

Accordingly, worldwide, there is strong interest in developing and providing physical activity and exercise interventions that help people of all age groups stay physically active, and preserve health and physical and mental resilience. Each decade has seen its trends. Among these, specifically yoga exercise has found its way into many exercise routines. Considering the last 10 years, there has been a constant increase of people, who self-report to practice yoga regularly as a weekly leisure time activity or as a health routine (e.g., Park et al., 2015; CompareCamp.com, 2020). In line with these trends, yoga has been implemented as a complementary or adjuvant therapy, for example in the treatment of eating disorders, depression, post-traumatic stress disorder, or anxiety (e.g., Hendriks et al., 2017; Meister and Becker, 2018) to name but a few examples. Moreover, health medicals and health insurances have started incorporating yoga in their exercise recommendations of health prevention.

In its traditional form, yoga is a philosophy and therefore much more than pure body exercise. In essence, yoga comprises several different aspects including mind and body exercises. From a scientific perspective, the majority of research investigating yoga interventions distinguishes four main categories or aspects that yoga exercise could comprise: (a) yoga postures, (b) breath control, (c) simple meditation, and (d) facets of mindfulness. Breath control and mindfulness techniques are often part of the yoga exercise itself and included in the yoga instructions (independently from separate meditation exercises). The attempt of these instructions is to facilitate body awareness during exercise by directing attention inwardly and away from the environment and keep an internal body focus during exercising rather than an external body focus on outward appearance (Ross and Thomas, 2010; Govindaraj et al., 2016). Western yoga interventions most frequently investigated in scientific studies so far focus on these four basic aspects, i.e., the practice of physical yoga postures in combination with controlled breathing, mindfulness instructions and sometimes, simple mediation exercise on top.

A recent review summarizing the results of studies that included yoga interventions with integrated practice (posture, breathing, mindfulness, and mediation exercise) found that yoga is equal or even superior to physical exercise interventions in improving health (Ross and Thomas, 2010). The review included 61 studies with a yoga pre-post design. Most of the studies were without a control group (*n* = 30). Some of them used a wait list control group (*n* = 16). Some studies compared the yoga intervention to an exercise intervention (*n* = 35). The studies investigated healthy study samples, or investigated patient populations diagnosed with NCDs. The outcome measures encompassed a broad range of health measures. The measures ranged from psychological self-report tools for mood/emotion, perceived stress, quality of life, pain, and fatigue assessment to the psychophysiological/psychobiological assessment of measures of functional fitness such as cardiovascular activity and heart rate variability (HRV), stress hormones (e.g., cortisol), blood glucose, cholesterol, energy expenditure, or VO2max (maximal oxygen uptake/consumption) as a measure of physical aerobic fitness. Yoga was effective in nearly all psychological measures and in nearly all physiological measures except improvement in aerobic fitness (e.g., VO2max, energy expenditure) when compared to exercise interventions (Ross and Thomas, 2010). Therefore, yoga interventions are not necessarily the primary choice for promotion of aerobic fitness compared to physical aerobic exercises such as running, cycling, or swimming. However, due to the suggested positive effects of yoga on well-being, on cardiac activity, the autonomous (ANS) and central nervous system (CNS), yoga interventions could be an ideal alternative to PA interventions for improving mind-body interactions and for improving resilience to physical and mental external or internal stressors.

### Yoga, Heart Rate Variability, and Neurovisceral Integration as Mind-Body Pathway

In the literature, changes in HRV have been consistently reported (Tyagi and Cohen, 2016). HRV describes the temporal variance between consecutive heartbeats in milliseconds (ms). This temporal variability is primarily under the control of the ANS including parasympathetic activity (PNS) mediated via the *N. vagus* and cardiac variations triggered by the sympathetic nervous system (SNP). Due to this relationship, HRV measures are promising and robust indicators of a person’s physical capacity to respond and adapt to mental, bodily (somatovisceral, interoceptive, and proprioceptive) and external (physical, sensory) stressors. In line with this assumption, cardiac autonomic activity is controlled by higher-order brain regions of the so-called CAN (central autonomic network; Benarroch, 1993). The CAN network acts as a brain-body hub for mind-body interactions. It comprises subcortical brain regions responsible for reflexive control of (cardiac) ANS activity as well as subcortical and cortical brain regions involved in top-down control of (cardiac) ANS activity. The CAN has reciprocal connections with other cortical and subcortical brain networks for the purpose of linking bodily sensation with emotional and autonomic responses at various system levels thereby allowing bodily homeostasis as well as reflexive and cognitively controlled physical adaption to internal and external emotional stressors (Thayer and Lane, 2000). It has been suggested, that HRV at rest as well as during or after recovery from mental or physical load can reflect the quality and effectiveness of this reciprocal connectivity between mental and bodily processes and the CNS (Benarroch, 1993; Porges, 2009). In fact, there is consensus in the literature that chronically reduced HRV at rest or during or after recovery from physical or mental load indicates reduced adaptability of the body to mental and external stressors (Fenzl and Schlegel, 2010). Furthermore, reduced HRV at rest or during mental or physical effort has been observed in patients suffering from NCDs such as diabetes or cardiovascular disorders. In addition, it has been observed in patients suffering from mental disorders such as depression, anxiety, or posttraumatic stress disorder; i.e., mental disorders, whose diagnostic key symptoms are characterized by changes in affect and mood and whose symptoms are accompanied by altered emotion processing and emotion regulation (Thayer and Brosschot, 2005).

Longitudinal studies suggest that yoga exercise improves HRV and cardiac autonomic regulation after a few weeks of practice pre- to post training (Tyagi and Cohen, 2016). In addition, single bouts of yoga may elicit an increase in HRV immediately after practice. This increase in HRV is mainly associated with an increase in vagal (parasympathetic) cardiac activity (Tyagi and Cohen, 2016). Changes in HRV after regular yoga exercise have also been confirmed in randomized controlled trial studies (RCTs), comparing yoga exercise or yoga breathing exercise to other mind-body exercises (Tai Chi) (Zou et al., 2018) or to physical exercises (Govindaraj et al., 2016) or control groups (e.g., Kuppusamy et al., 2020). Moreover, there is some evidence from meta- and review studies that yoga can alleviate acute and chronic stress, depressive and anxiety symptoms in certain patient groups compared to placebo controls or treatment as usual (Cramer et al., 2017; Vollbehr et al., 2018). Moreover, changes in mood and emotion processing on the one hand and in cardiac autonomic functioning as reflected in modulation of HRV on the other hand have been reported after weekly regular yoga practice (Tyagi and Cohen, 2016; Mocanu et al., 2018).

### Open Questions and Aim of the Present Study

Consequently, it could be and has been speculated that many of the effects of yoga on health and well-being reported in the literature could be explained by the neurovisceral integration principle outlined above (Brown and Gerbarg, 2005). However, still, critical questions to answer are, whether changes in autonomic functioning as reflected in the modulation of HRV and changes in mood and emotion processing occur together instantaneously after already a single session of yoga exercise and whether effects are the same when yoga postures are practiced with or without breath control and mindful body awareness. So far, there are only some studies allowing an answer to these questions. As far as mood and emotion processing is concerned, the majority of yoga intervention studies focused on self-report measures (Narasimhan et al., 2011) and concentrated on comparisons between yoga practitioners vs. control groups (Froeliger et al., 2012; Fiori et al., 2017). The results of these studies provided tentative support for the thesis that regular yoga practice promotes adaptive emotion processing by increasing a self-centered perspective during emotion processing. A self-centered perspective should facilitate self-referential processing and attention to one’s own emotions while at the same time enhancing feelings of compassion and social connectedness (Fiori et al., 2017). However, as pointed out by the existing meta- and review studies, evidence is so far limited. Studies that investigated immediate pre- to post effects of yoga practice on emotion processing in experimental designs, that allow for controlled assessment of changes in emotion processing and in HRV modulation either in the laboratory or by means of ambulatory assessment are so far largely lacking in the literature.

Regarding beat-to beat fluctuations in HRV, these are not independent from breathing. The interval between heartbeats (R-R interval) is shortened during inspiration and it is prolonged during expiration. This dependency is known as respiratory sinus arrhythmia (RSA). At rest, and with normal breathing rate, RSA is thought to be mainly under parasympathetic control, and this is reflected by HF-HRV modulation within the high-frequency range (0.15–0.40 Hz). Specific yoga breathing techniques as well as voluntarily controlled slow breathing (e.g., breathing at six cycles/minute) as well as specific biofeedback training such as resonant frequency biofeedback training can influence measures of HRV and HRV modulation (Lehrer et al., 2000; Zaccaro et al., 2018; Kuppusamy et al., 2020). Breathing and cardiac activity are both under the control of the ANS and the CAN. Furthermore, rhythmical muscle tension at 0.1 Hz (Lehrer et al., 2009), and combining and aligning paced breathing with dynamic muscle contraction (e.g., paced breathing synchronized with alternating contraction vs. neutral control, Chin and Kales, 2019) has been shown to affect autonomic nervous system reactivity and increase parasympathetic, vagal activity as indicated by high-frequency measures of HRV. Moreover, instructing participants to perform the body postures together with breathing during yoga should increase body awareness (Daubenmier et al., 2012), especially when related with mindfulness instructions directing attention to breathing and the body (Sze et al., 2010; Telles et al., 2011, 2014). Consequently, more pronounced changes in HRV, body awareness and self-referential emotion processing could be expected when yoga postures are exercised together with breathing and mindfulness body focus instructions compared to when yoga postures are exercised without such explicit breathing and mindfulness body focus instructions.

The aim of the present study was to test these hypotheses of a relationship between yoga and changes in HRV and emotion processing after a single session of yoga exercise. In addition, the present study compares the effects of exercising the yoga postures with and without breathing instructions and mindful body awareness. As outlined above, breathing and mindfulness are two essential aspects of yoga. To this end, two experimental groups were included in the study whose participants performed the same yoga exercises once with controlled breathing and mindfulness instructions and once without breathing and mindfullness instructions. To induce and examine changes in emotion and in self-other referential processing, an experimental standardized emotion task was used to investigate if yoga exercise increases self-referential and emotion processing when compared pre to post practice. Furthermore, awareness of bodily signals was assessed experimentally and by means of standardized questionnaires, and in addition, interindividual differences in empathy and self-other referential emotion processing were controlled via self-report. To avoid age and sex specific confounds the study focused on healthy adults, university students and women only.

## Materials and Methods

### Participants

*N* = 34 university students, all women (*mean age*: 22.87 years, *SD* = 4.0; *age range*: 18–39 years) participated in the study. Inclusion criteria for participation were an age of 18 years or above, being native speaker of German language, no current injuries, not being pregnant and no history of cardiovascular or other mental, neurological, or somatic diseases. All participants were questioned about their regular exercise and physical activity habits using a short exercise assessment questionnaire. This revealed that *n* = 23 participants (76.7%) of the participants reported to engage in at least one type of physical activity (e.g., jogging, swimming, cycling, dancing, team sports, martial arts, strength training, balance, or gymnastics) for at least once a week for at least 6 months, on average, for about *M* = 9.7 years (*SD* = 5.68). In addition, *n* = 5 (16.7%) of the participants reported to have some experience with yoga, practicing yoga once a week during the last 6 months, on average for *M* = 3.4 years (*SD* = 3.91). Thus, the majority of the participants (*n* = 25, 83.3%) were naïve to yoga practice. The study was advertised via flyers and emails on the university campus system. During study advertisement and during the recruitment of the participants there was no mention about yoga, body awareness, breathing or emotion processing, excluding any sampling bias by avoiding buzzword related anticipatory effects that could have biased the participants’ expectancies about participation or influence selection to particular interested or experienced individuals or groups.

#### Ethics Statement

Participation in the study was voluntary, participants were instructed in detail that they could reject from participation throughout the study without negative consequences. All participants gave written informed consent. They were debriefed about inclusion and exclusion criteria and the procedure of the study design prior to participation. Only participants who fulfilled the inclusion criteria stated above and who gave their written informed consent could take part in the study.

### Study Design

The participants were randomly assigned to two exercise groups. Serial assignment (A-B) was used in accord with the criteria of a RCT. Serial assignment is giving participants the equal chance to be assigned to one of the two exercise groups. Serial random assignment led to random assignment of the *n* = 5 participants who self-reported experience with yoga practice to the two exercise groups (i.e., *n* = 2 of these participants were in exercise group 1) and three of these participants were randomly assigned to the exercise group 2. The two groups performed the same exercises, however, with different instructions (see Supplementary Table 1). The experimental group 1 (exercise group 1) performed the exercises with instructions to exercise any movement with heightened body awareness and breathing control. The experimental group 2 (exercise group 2) performed the exercises without explicit instructions of breathing control or internal body focus. The exercises consisted of the same exercises in each exercise group. The exercises selected in both groups were taken from a pool of yoga exercises according to the following criteria: (a) the exercises should be easily performed by yoga or exercise novices who do not engage in regular exercise or sports, (b) all exercises should be simple and to be carried out with little effort to ensure alignment with breathing and body focus instructions (group 1), (c) the exercises should include only postures with no injury risk and guided by an instructor. Yoga postures with heavy movements, strong stretches or reverse postures were not included to avoid risky movements and difficulties in breathing. The individual exercises lasted on average 3 min. The whole exercise session lasted 30 min for both groups. A description of the exercises is provided in Supplementary Table 1 for an overview.

#### Procedure

##### Interventions and Exercise Groups

In both exercise groups, the exercises were instructed by the same female instructor of the same age of the participants. In the exercise group 1, the performance of the individual yoga exercises was instructed in such a way that the attention of the participants was repeatedly drawn to their breath. As shown in Supplementary Table 1, the participants were asked to feel into their body, be aware of the difference between tension and relaxation, attend to their breath and try to align their breath to the individual movements of the postures as far as they could. Of note, no particular slow breathing yoga exercise or resonance breathing of 0.1 Hz was chosen or included. The movements were carried out with eyes closed to facilitate concentration on the body. In the exercise group 2, performance of the individual exercises was also guided by verbal instructions; however, as shown in Supplementary Table 1, no particular instruction was given to pay attention to breathing or to internal bodily processes, or of being aware of tension vs. relaxation or of controlling breathing. In the exercise group 2, the individual exercises were introduced as stretching exercises, carried out with eyes open. Before the start of the exercises, both exercise groups performed a 3 min period of relaxation and finished the exercise session by a recovery period of the same length.

##### Assessment Tools

###### Questionnaires

Upon arrival at the lab, participants were questioned about their age, health and exercise and PA routines including current and past injuries and history of disorders (see section “Participants”). Next, the participants filled in a number of questionnaires. The questionnaires included the German version of the positive and negative affect scales of the PANAS (Watson et al., 1988) for current mood state assessment. In addition, the German version of the Multidimensional Assessment of Interoceptive Awareness Questionnaire (MAIA, Mehling et al., 2012) was used for the assessment of eight different facets of habitual body awareness such as “noticing,” “listening to the body,” “attention regulation,” “self-regulation,” “not-worrying,” or “trusting.” The Saarbrückener Personality questionnaire [German version of the Interpersonal Reactivity Index (IRI), Davis, 1980] was included for the assessment of empathy and self-other referential processing including the subscales “perspective taking,” “fantasy,” “empathic concern,” and “personal distress.”

###### Experimental Paradigms

**FIGURE 1:**
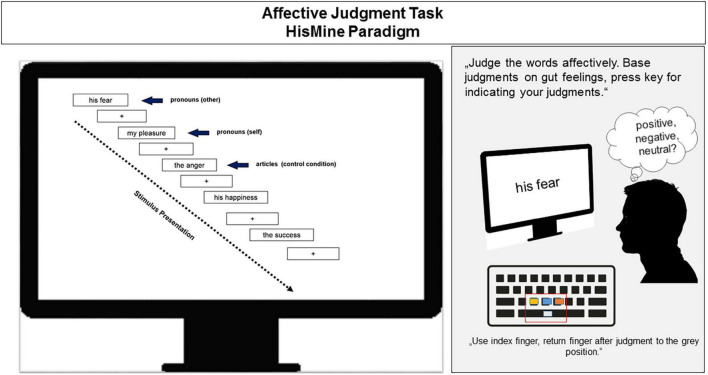
llustration of the affective HisMine paradigm (Herbert et al., 2011). For details, please refer to the text.

The affective HisMine Paradigm (aHMP; e.g., Herbert et al., 2011) was used as affective judgment task for the investigation of self- vs. other referential emotional processing. The affective HisMine Paradigm was presented on a computer screen. In the aHMP (see Figure 1 for an illustration), the reader views a series of pronoun-noun pairs preselected to comprise nouns of positive or negative emotional content such as “fear” or “joy” or of neutral content (e.g., “cloths,” “books,” or “furniture”), all matched on average for emotional valence and emotional arousal, and linguistic dimensions such as word-length. The nouns are paired with self-referential or other-referential pronouns of the first or third person “my” or “his” or with articles having no person reference (“the”). Participants were instructed that pronoun-noun pairs can belong to the self and refer to the reader’s own emotions or reflect objects and feelings of a third person or describe feelings or objects in general (article-noun pairs). For each trial and word pair, participants were instructed to make a quick affective valence judgment (like, dislike, and neither/nor), which they should base solely on their gut feelings and indicate their judgment by pressing one of three preselected keys on the keyboard (positive/like, negative/dislike, or neutral/neither-nor). In total, 108 pronoun-noun pairs were presented, 18 pairs per emotion category (positive, negative, and neutral) and reference category (self, other, and article). The word pairs were presented for 5 s each and followed by a randomly jittered inter-stimulus-interval in which a fixation cross was shown for about 1–1.5 s before the onset of the next trial. The affective task was presented before (affective task 1) and after the exercise session (affective task 2). Because in the affective judgment task, the words were presented pseudorandomly and each participant was given his/her own stimulus presentation randomization, carry over or memory effects across trials and across repeated exposure pre- to post exercise are avoided. For an illustration of the affective HisMine Paradigm, see Figure 1.

**FIGURE 2:**
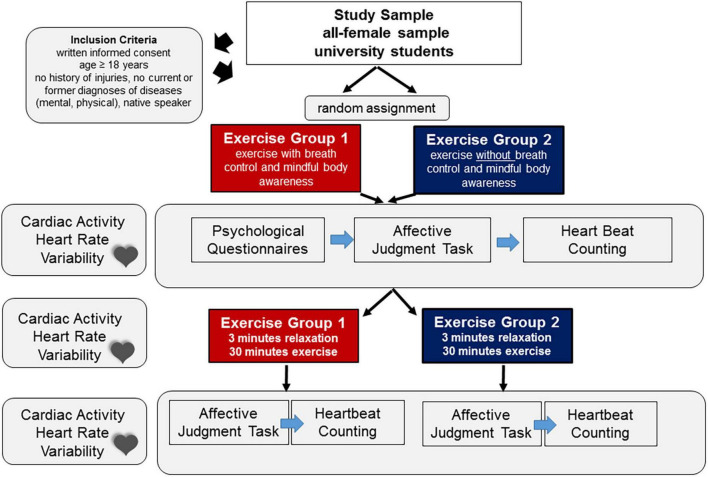
Overview of the study design.

In addition, participants completed the heartbeat-counting task (Schandry, 1981), one of the most widely used (yet not uncritized, e.g., Brener and Ring, 2016) measure for assessing accuracy and awareness of internal bodily signals (here heartbeats). Instructions followed standard guidelines, i.e., participants were instructed to count their heartbeats in a certain time interval. The length of the time intervals was not communicated to the participants and randomly presented to the participants. The individual length of the counting intervals were of 25, 45, or 35 s durations and each interval was followed by a break of about 20 s. Participants were instructed by the experimenter when to count and when to stop and had to report their counts to the experimenter. Actual heart rate was recorded to calculate measures of accuracy (see in section “Data Analysis”). Participants had to rest in supine position, with legs and arms uncrossed. Akin to the affective judgment task, the heartbeat-counting task was presented twice to the participants, once before the exercise session and once immediately after the exercise session (see Figure 2).

###### Cardiac Activity

Cardiac activity was assessed continuously across the different tasks and during the exercise session using the Polar watch RS800CX^1^. The Polar watch RS800CX measures inter-beat variations (IBI) via elastic electrode sensors strapped around the chest and fixed below the chest muscles. Data recording corresponded to one millisecond resolution (corresponding to a sampling rate of 1,000 Hz). Data were exported as IBIs in ms. Participants of the two exercise groups were invited to the lab individually from Monday to Friday from 9:00 a.m. to 10:30 a.m., from 10:30 a.m. to 12:00 a.m., from 1:30 p.m. to 3:00 p.m. and from 3:00 p.m. to 4:30 p.m. No appointments were made before 9:00 a.m. or 12:00 a.m., respectively. Participants were asked to wear comfortable clothing at their individual testing day and not eat a large meal or drink beverages with caffeine or smoke immediately before the exercise to avoid confounds in psychophysiological measures. Hormonal status of the participants and menstrual cycle can affect HRV measures (Sims et al., 2021). Hormonal status and menstrual cycle were not controlled in the present study due to random assignment of the participants to the two groups, possibly excluding bias or differences between participants regarding menstrual cycle or any other confounding variables.

For an overview of the complete study design, please see Figure 2.

## Data Analysis

### Cardiac Activity – Heart Rate Variability

#### Indices and Preprocessing

Heart rate variability was calculated from the raw data (inter-beat intervals) exported from the Polar watch RS800CX. The raw data were recorded continuously during the different tasks and assessment periods. The data epochs of interest comprised for each subject the recordings during the affective judgment tasks before and after the exercise intervention (10 min each) as well as the recordings during the 30 min exercise intervention. The data recording during questionnaire assessment (10 min) served as starting baseline (see section “Results”) and data recording during the 3 min relaxation period at the start of the exercise session served as resting baseline for additional control of group comparisons. The data recordings during the heartbeat-counting tasks (pre- and post-exercise) were used for calculation of the heartbeat-counting indices. No HRV measures were extracted from these intervals. The ARTiiFACT software (Kaufmann et al., 2011) was used for automated data preprocessing and calculation of HRV indices. ARTiiFACT is a free open source software tool for heart rate artifact processing and HRV analysis. The software accepts data from real ECG recordings or IBI data collected from digital devices such as the Polar watch RS800CX. Based on the imported data files, time-domain and frequency-domain HRV measures can be obtained from the software and different modes of artifact preprocessing including artifact rejection or correction can be chosen and combined. In the present study, artifacts were preprocessed by cubic spline interpolation as the standard procedure for the replacement of missing or artificial IBIs (Kaufmann et al., 2011). All data sets were treated by the same software default values and the same criteria were applied for data preprocessing and rejection of noisy raw data. Especially for the recording during the exercise, care was taken to remove movement artifacts. HRV guidelines suggest a minimal length of 5 min recording to be the reliable gold standard for the analysis of time-domain and frequency-domain HRV measures (Task Force of the European Society of Cardiology, 1996; Shaffer and Ginsberg, 2017). However, even shorter recording times of about 2 min can be used without confounding or biasing frequency-domain HRV or some of the time-domain HRV measures (Bourdillon et al., 2017).

In line with HRV guidelines, time-domain and frequency-domain HRV measures were analyzed. Changes in the high frequency and low frequency HRV components are reported as HF- and LF-HRV in normalized units, HF-HRV (n.u.) and LF-HRV (n.u.), respectively. The HF- and LF-HRV components are derived from the spectral frequency spectrum after Fast Fourier Transformation. The standard default software values for the bandwidth of the two frequency-domain components were used for decomposition including the domain of 0.15–0.4 Hz for the HF-HRV component and the domain of 0.04–0.15 Hz for the LF-HRV component, respectively. Among the HRV frequency-domain measures and when measured at rest, the HF-HRV component has proven to most reliably reflect changes in cardiac activity related to parasympathetic vagal activity. At rest, the frequency domain of the HF-HRV component lies in the range of normal breathing. However, exercise and certain breathing maneuvers can lead to a shift in frequencies of the HF-HRV component. Therefore, beat-to beat fluctuations in synchrony with breath as reflected in respiratory sinus arrhythmia, RSA (logRSA) was also estimated from the artifact corrected IBI time series data using special software (Allen, 2002; http://apsychoserver.psych.arizona.edu/MetricsSoftware.htm). LogRSA was calculated for the exercise session and was compared between the two exercise groups.

Two HRV time-domain measures were analyzed: Root Mean Square of Successive Differences (RMSSD) in ms and pNN50. As a HRV time-domain and variability measure, RMSSD (ms) best reflects immediate adaptability of the heart to rapid changes elicited by external and internal stressors. Unlike the HF-HRV component, RMSSD is less affected by changes in respiration (Shaffer and Ginsberg, 2017). Akin to the RMSSD, the pNN50 is an index of cardiac variability (Shaffer and Ginsberg, 2017). The pNN50 characterizes the proportion or percentage (%) of pairs of consecutive inter-beat (RR) intervals that divided by the total number of NN (RR) intervals are more than 50 ms apart. Together with the RMSSD measure, pNN50 is an index of the quality of cardiac chronotropic adaptability. Given its sensitivity to conditional changes such as body posture, temperature, etc., the pNN50 was compared only during the affective judgment task having the same standardized physical recording (e.g., same supine position, same recording length).

### Experimental Paradigms

#### Emotion Processing: Affective Judgments of Self-Other-Referential Emotion Stimuli

The affective judgment task, comprising self- and other-referential emotional stimuli (see Figure 1) allows analysis of two outcome measures. First, speed of access to one’s gut feelings and second, the accuracy of affective judgments. Reaction times in ms are indicators of processing speed. The number of times the participant’s judgment is congruent with the individual valence of the word reflects the accuracy of affective judgments. Accuracy (number of valence congruent answers in %) and speed of affective judgments (in ms) were analyzed for the word-pairs that vary in emotional valence (positive/pleasant, negative/unpleasant, or neutral) and in self-other reference [self, other, and no person reference (articles)] resulting in 3 × 3 repeated measures design.

#### Internal Bodily Signals

The heartbeat-counting task allows calculation of a performance score as absolute difference between the number of actual heartbeats (recorded by the recording device) and the participant’s reported heartbeats (documented by the experimenter). The performance score varies from zero to one. The standardized quantitative score reflects the participants’ estimate of confidence in the appraisal of felt heartbeats.

### Questionnaires

The PANAS (Watson et al., 1988), MAIA (Mehling et al., 2012), and SFP/IRI (Davis, 1980; Paulus, 2009) questionnaires provide sum scores for total scores and individual subscales. The sum scores were correlated with HRV and affective measures (reaction time, accuracy) to explore potential relationships between psychological measures and experimental measures prior to and after the exercise session.

### Statistics and Data Exclusion

Of the *N* = 34 participants, data of *n* = 2 participants per exercise group had to be excluded due to technical failure in signal recording. Data of *N* = 30 participants could be included in the statistical analysis. The data were statistically analyzed by repeated measures analysis of variance (ANOVA). Mean heart rate (mean HR in beats per minute) as well as HRV frequency-domain (HF n.u.; LF n.u.) and time-domain measures (RMSSD, pNN50) were analyzed during the exercise session as well as during the affective judgments task pre-to post exercise. Repeated ANOVAs were calculated comprising the factor “condition” (affective task 1, exercise, and affective task 2) as within-subject factor and “group” as between subject factor. The factor “condition” included three levels (task 1, exercise, and task 2). The three levels allow (a) to investigate changes in cardiac activity (HR) and variability (HRV) in the affective task as a function of the exercise session (pre to post) and (b) to determine whether the exercise as a physical stressor was successful in changing mean HR and HRV measures during exercise as compared to a pre-exercise affective task baseline. To control for differences between the exercise groups at the beginning of the exercise session, mean HR and HRV were compared during the 3 min relaxation baseline. Speed and accuracy of affective judgments were analyzed with repeated measures ANOVAs, each containing the within-subject factors “time” (pre-exercise vs. post-exercise), “emotion” (positive, neutral, and negative), and “reference” [self, other, and no person reference (articles)] and the between-subject factor “group” (exercise group 1 vs. exercise group 2). Power-estimation for repeated measures with between-within interactions and an effect size of *f*(U) = 0.5 (corresponding to a partial eta^2^ of 0.2) for testing differences between the two exercise groups, e.g., in HRV measures pre-, during, and post exercise (2 × 3 design) suggest a total sample size of *N* = 36, and a total sample size of *N* = 22 for the factorial design of the affective judgment task (2 × 3 × 3 × 2) and an effect size of *f*(U) = 0.3.

Given the small sample size of the final sample comprising only *N* = 15 participants per group, and the ANOVA design, examination of variance was included in *a priori*-testing (e.g., Levene test). When sphericity was not met in repeated measures ANOVAs containing more than two levels, *p*-values are reported corrected according to Greenhouse Geisser. *Post-hoc* testing of significant main effects and significant interaction effects were performed with contrast tests. Pearson’s *R* was used for correlations between questionnaire and HRV and affective measures and for assessment of cross-correlations between time-domain and frequency-domain HRV measures, including RMSSD, pNN50 and HF-HRV (n.u.), respectively. The corresponding statistical tests are reported together with their corresponding test value (*F*-statistics) and *p*-values (Greenhouse Geisser corrected, where appropriate) in the section “Results.”

To explore whether the self-reported previous yoga experience of *n* = 5 participants might constitute a confounding factor, exploratory analysis were included that descriptively compared the degree to which self-report measures and measures of cardiac activity differed for these individuals compared to those individuals who did not report any previous yoga experience. In addition, the statistical analysis of cardiac activity across experimental conditions in the two exercise groups was performed without the *n* = 5 participants reporting previous yoga experience.

## Results

### Cardiac Activity and Heart Rate Variability

#### Mean Heart Rate

**FIGURE 3:**
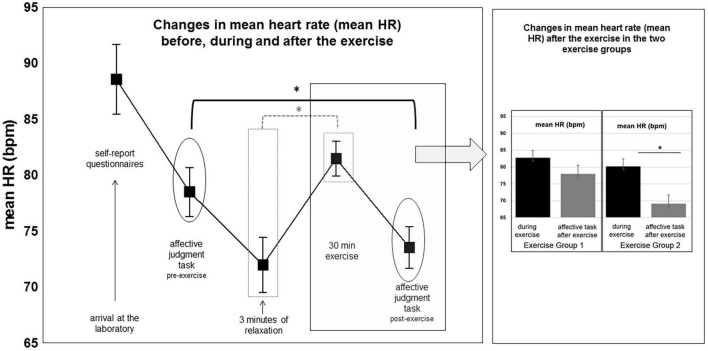
Changes in mean HR across the different experimental conditions. For details, see text.

Mean HR showed a significant main effect of the within-subject factor “condition” [*F(*2,56) = 22.93, *p* < 0.001], and a significant interaction of the factors “condition” × “group” [*F*(2,56) = 4.72, *p* = 0.02]. Planned contrast tests showed the following changes in mean heart rate (see Figure 3): mean HR did not significantly increase during the exercise session relative to during the affective task pre-exercise [*F*(1,28) = 4.02, *p* = 0.055]. However, mean HR recorded during the affective tasks was significantly lower after the exercise session as compared to before the exercise session [*F*(1,28) = 20.64, *p* < 0.001]. This pre-post difference was found it both exercise groups and was also significant when the two exercise groups were analyzed separately [exercise group 1: *F*(1,28) = 10.22, *p* < 0.005; exercise group 2: *F*(1,28) = 10.91, *p* < 0.005]. In addition, mean HR was significantly lower during the affective task post-exercise as compared to during the exercise sessions [*F*(1,28) = 79.35, *p* < 0.001]. Again, this was found, when the two exercise groups were compared separately [exercise group 1: *F*(1,28) = 14.49, *p* < 0.001; exercise group2: *F*(1,28) = 77.29, *p* < 0.001]. However, the relative decrease in mean HR (from during the exercise to during the affective task post-exercise) was significantly more pronounced in the exercise group 2, who performed the postures without instructions of breath control and mindfulness instructions [*F*(1,28) = 12.42, *p* = 0.0015]. Changes in mean heart rate are illustrated in Figure 3.

#### Heart Rate Variability

##### Frequency-domain

**FIGURE 4:**
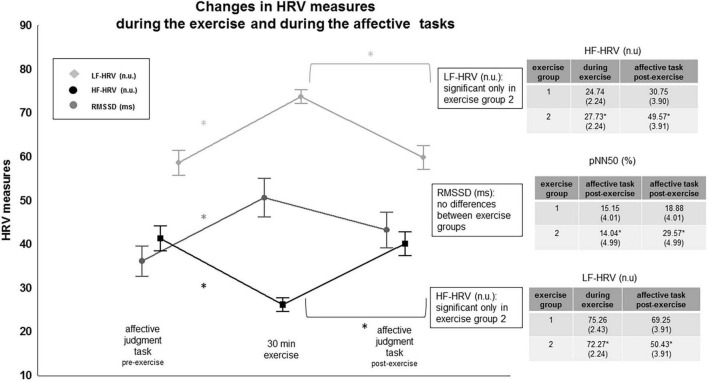
Changes in HRV frequency-domain and HRV time-domain measures. “*” indicates significance. For details, see text.

The HF-HRV component HF (n.u.) showed a significant main effect of the within-subject factor “condition” [*F*(2,56) = 22.06, *p* < 0.001], and a significant interaction of the factors “condition” × “group” [*F*(2,56) = 6.99, *p* = 0.003], see Figure 4. Comparisons between conditions yielded a significant decrease in the high frequency component (HF n.u.) during the exercise session compared to during the affective task pre-exercise [*F*(1,28) = 54.97, *p* < 0.001], and a significant increase during the affective task post-exercise as compared to during the exercise session [*F*(1,28) = 28.33, *p* < 0.001]. Planned contrast comparisons showed that this increase in HF-HRV (n.u.) from during the exercise to during the affective task post-exercise was significant in the exercise group 2, who performed the yoga postures without instructions of breath control and mindfulness instructions [*F*(1,28) = 34.85, *p* < 0.001]. It was not significant in the exercise group 1, who performed the yoga postures with instructions of breath control and mindfulness instructions [*F*(1,28) = 2.63, *p* = 0.10]. The HF-HRV (n.u.) component did not differ between the two exercise groups during the exercise session nor during the affective task pre-exercise (all *p* > 0.1). The low frequency LF-HRV (n.u.) component showed the reverse effects of the high frequency component for the main effect of the within-subject factor “condition” [*F*(2,56) = 22.21, *p* < 0.001], and the interaction of the factors “condition” × “group” [*F*(2,56) = 6.98, *p* = 0.019], see Figure 4. Contrast tests revealed a significant increase in LF-HRV (n.u.) from the affective task pre-exercise to during the exercise session irrespective of “group” (exercise group 1 and exercise group 2, respectively), [*F*(2,56) = 54.96, *p* < 0.001]. The LF-HRV (n.u.) was significantly lower during the affective task after the exercise only in exercise group 2, [*F*(2,56) = 34.85, *p* < 0.001]. Both exercise groups did not differ in the LF-HRV (n.u.) component during the exercise session or during the affective task pre-exercise (all *p* > 0.1).

#### Heart Rate Variability

##### Time-domain

The HRV time-domain measure RMSSD showed no significant interaction effect, but a significant main effect of “condition” [*F*(1,28) = 6.49, *p* = 0.002], see Figure 4. RMSSD scores increased significantly during the exercise session compared to during the affective task before the exercise session [*F*(1,28) = 9.92, *p* = 0.005]. Mean RMSSD scores were significantly higher during the affective task after the exercise than during the affective task before the exercise [*F*(1,28) = 17.22, *p* = 0.001]. Mean RMSSD scores during the exercise session and mean RMSSD scores during the affective task after the exercise session did not differ: the scores were significantly higher than the mean RMSSD scores obtained during the affective task before the exercise session.

Following recording guidelines, pNN50 was compared during the affective tasks (pre-post exercise) only, to keep physical recording conditions similar. The repeated measures ANOVA showed a significant main effect of “condition” [*F*(1,28) = 8.82, *p* = 0.006] and a significant interaction of the factors “condition” × “group” [*F*(1,28) = 13.40, *p* < 0.001]. Contrast tests revealed a significant difference between the two exercise groups from pre- to post exercise. The pNN50 score during the affective tasks was significantly higher post-exercise compared to pre-exercise in the exercise group 2 only, [*F*(1,28) = 21.99, *p* < 0.001]. The exercise group 1 did not differ in this measure during the affective task pre- to post exercise. The pNN50 during the affective tasks post-exercise was also significantly higher in the exercise group 2 than in the exercise group 1 [*F*(1,28) = 5.96, *p* = 0.02], whereas the two exercise groups did not differ in this measure during the affective task before exercise (*p* > 0.1).

#### Changes in Heart Rate Variability Measures During Baseline

Heart rate variability measures were also analyzed during the questionnaire period preceding the affective task 1, pre-exercise (10 min) and the 3 min relaxation period following the affective task and preceding the exercise session. As shown in Table 1, the two exercise groups did not differ significantly from each other in the analyzed cardiac measures [mean HR, HF-HRV (n.u.), LF-HRV (n.u.), RMSSD, pNN50, respectively], neither during the questionnaire assessment nor during the 3 min relaxation period.

**TABLE 1:** Changes in HRV measures during the two baseline recordings comprising the questionnaire period preceding the affective task 1, pre-exercise (10 min) and the 3 min relaxation period following the affective task and preceding the 30 min exercise session.

HRV measures at baseline							

Exercise group1^+^	Measure	Mean	SE	Exercise group2^+^	Measure	Mean	SE
Self-report	mean HR	88.41	4.39	Self-report	mean HR	88.72	4.39
Self-report	RMSSD	31.47	4.84	Self-report	RMSSD	33.08	4.84
Self-report	pNN50	13.27	3.73	Self-report	pNN50	13.03	3.73
Self-report	LF-HRV (n.u.)	69.63	2.61	Self-report	LF-HRV (n.u.)	67.03	2.61
Self-report	HF-HRV (n.u.)	30.37	2.61	Self-report	HF-HRV (n.u.)	32.97	2.61
Relax	mean HR	76.35	3.46	Relax	mean HR	67.61	3.46
Relax	RMSSD	53.07	9.36	Relax	RMSSD	70.86	9.36
Relax	pNN50	27.28	5.86	Relax	pNN50	37.37	5.86
Relax	LF-HRV (n.u.)	57.62	6.47	Relax	LF-HRV (n.u.)	52.15	6.47
Relax	HF-HRV (n.u.)	42.38	6.47	Relax	HF-HRV (n.u.)	48.24	6.47

**Respiration triggered cardiac activity during exercise**							

Exercise group1^+^	Measure	Mean	SD	Exercise group2^+^	Measure	Mean	SD
During exercise	logRSA	6.32	0.56	During exercise	logRSA	6.17	1.04

*Lower column: LogRSA: respiration-related normalized beat-to-beat fluctuations, i.e., respiration triggered changes in HRV during the exercise in the exercise group 1 and the exercise group 2.*

*SE: standard error; self-report: during fillingout of the questionnaires; relax: during the 3 min relaxation period. ^+^ no significant differences between the two exercise groups. SD: standard deviation, ^+^ no significant differences between the two exercise groups.*

#### Respiration Triggered Heart Rate Variability During Exercise

LogRSA as a measure of respiration-related normalized beat-to-beat fluctuations did not significantly differ (*p* = 0.67) between the two exercise groups during the exercise sessions despite a larger standard deviation in the exercise group 2, who practiced without breathing instructions and mindful body instructions compared to exercise group 1, who practiced with breath instructions and mindful body instructions, see Table 1.

### Experimental Paradigms

#### Emotion Processing: Affective Judgments of Self-Other-Referential Emotion Stimuli

**FIGURE 5:**
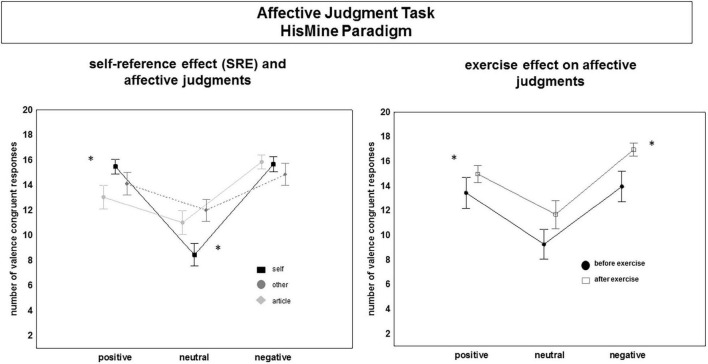
Changes in affective judgments (accuracy) before and after the exercise session. Main effects and interaction effects. “*” indicates signficance. For details, see text.

Accuracy (number of valence congruent responses) showed a significant main effect of the factor “time” [*F*(1,28) = 18.74, *p* < 0.002]. Overall, accuracy of affective judgments was higher after the yoga exercise compared to pre-exercise. In addition, accuracy varied as a function of the factor “emotion” [*F*(2,56) = 43.71, *p* < 0.001]. Valence congruent responses were higher for negative and positive than for neutral words and this was modulated by the self-other reference of the words as well as by the factor “time” as was indicated by the significant interaction effects of “emotion” × “reference” [*F*(4,112) = 25.80, *p* < 0.001] and of “emotion” × “time” [*F*(2,56) = 3.50, *p* = 0.037]. As shown in Figure 5, the number of valence congruent responses was significantly higher for self-referential positive words as compared to other-referential positive words [*F*(1,28) = 18.57, *p* < 0.002] or positive words without person reference [*F*(1,28) = 11.78, *p* < 0.002], irrespective of time (pre- vs. post-exercise). For neutral words, the number of valence congruent responses was lowest for self-referential neutral words as compared to other-referential neutral words [*F*(1,28) = 29.30, *p* < 0.001] or neutral words presented without person reference [*F*(1,28) = 56.67, *p* < 0.001]. For negative words, the number of valence congruent responses did not differ with self-other reference. However, when compared across time (pre- to post exercise), the accuracy of affective judgments and thus the number of valence congruent responses was significantly higher for negative, neutral and positive words, irrespective of the words’ self-other reference (all *p* > 0.05).

**FIGURE 6:**
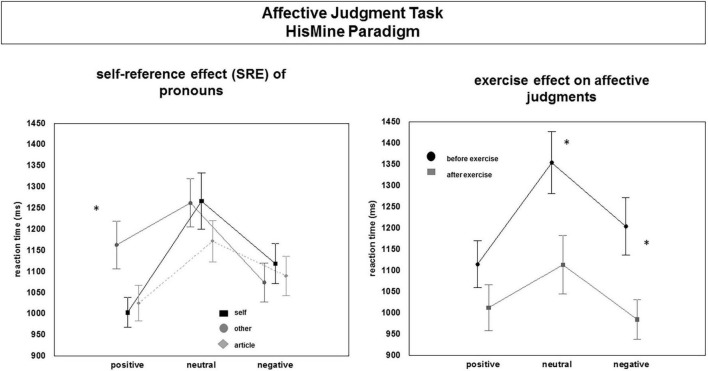
Changes in affective judgments (reaction times) before and after the exercise session. Main effects and interaction effects. “*” indicates significance. For details, see text.

Reaction time data showed a significant main effect of “time,” [*F*(1,28) = 71.35, *p* < 0.001]. Overall, reaction times were shorter post-exercise compared to pre-exercise. Moreover, the speed of affective judgments (reaction time) was significantly modulated by the “emotion” [*F*(2,52) = 23.24, *p* < 0.001] and by the self-other reference of the words [“reference”: *F*(2,56) = 13.94, *p* < 0.001] and by the interaction between the factors “emotion” and “reference” [*F*(4,112) = 11.46, *p* < 0.001]. In addition, there was a significant interaction between “emotion” × “time”: [*F*(2,56) = 12.79, *p* < 0.001]. Contrast tests showed significantly faster affective judgments for positive and negative as compared to neutral words. As shown in Figure 6, participants were faster in their affective judgments for self-referential positive words as compared to other-referential positive words [*F*(1,26) = 28.11, *p* < 0.001] or positive words having no person reference [*F*(1,28) = 4.38, *p* < 0.05]. Judging neutral words elicited the longest reaction times, especially affective judgments of self-referential and other-referential neutral words. Self-referential and other-referential negative words were judged as fast as negative words without self-other reference before the exercise session as well as after the exercise session. Before the exercise session, the participants were faster in their affective judgments for positive words compared to negative and neutral words, [*F*(1,28) = 40.82, *p* < 0.001], and [*F*(1,28) = 11.91, *p* = 0.002]. After the exercise session, participants were faster in their affective judgments for negative and positive words compared to neutral words, [*F*(1,28) = 19.32, *p* < 0.001 and *F*(1,28) = 8.61, *p* = 0.006].

#### Internal Bodily Signals

##### Heartbeat-Counting Task

Comparison of the performance in the heartbeat-counting task was analyzed within a repeated measures ANOVA design comprising the within-subject factor “time” (pre- to post exercise) and “group” as between-subject factor. This revealed a significant effect of “time” [*F*(1,28) = 13.65, *p* < 0.002], pre-exercise: 0.59 (*SD* = 0.16) vs. post-exercise: 0.66 (*SD* = 0.19). Overall, participants came closer in their estimations of counted heartbeats to actual heartbeats after the exercise session. The two exercise groups did not differ in their performance in the task pre-exercise. The exercise group 2, who exercised without instructions of breath control and mindfulness instructions were more accurate pre to post exercise (score: 0.73) as compared to the exercise group 1, who exercised with instructions of breath control and mindfulness instructions (score: 0.62). The interaction of “time” × “group,” however, was not significant and showed only a trend toward significance [*F*(1,28) = 3.92, *p* = 0.058].

### Interindividual Differences

#### Self-Report Questionnaires

**FIGURE 7:**
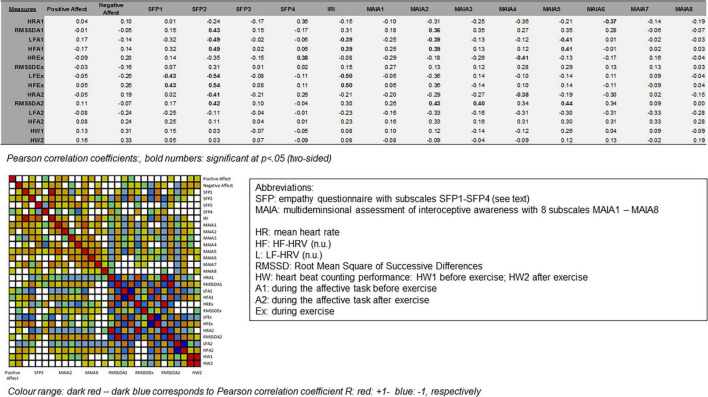
Correlation analyses. Plots and tables. For details, see text.

Correlation analyses revealed only a few significant positive and negative correlations between the questionnaires assessing self-reported differences in emotion processing, awareness of bodily signals, HRV indicators (e.g., HF-HRV, RMSSD), affective measures (reaction time, accuracy) and heartbeat counting performance. HRV measures were, however, significantly correlated within and across experimental conditions with each other, thus showing high reliability of HRV measures across tasks. As illustrated in the correlation plots in Figure 7, the few correlations between questionnaire measures and HRV measures suggest that trait-like interindividual differences did not significantly correlate with HRV measures. Moreover, there were no significant correlations with affective measures pre- to post exercise.

#### Exploratory Analysis

##### Previous Yoga Experience

From the final all-female sample, *n* = 5 participants reported to have practiced yoga in the past, of these participants, *n* = 3 reported practicing for 1 year, *n* = 1 reported practicing for 4 years, and *n* = 1 reported practicing already for 10 years. Because previous studies (see section “Introduction”) have shown that yoga practice can have an impact on various health dimensions (including body focus, breath control, and cardiac activity, practiced HF-HRV activity in particular), the following comparisons were tested. First, it was explored if the *n* = 5 participants obtained higher scores in the self-report measures of empathy and habitual body awareness. Next, it was explored if the *n* = 5 participants differed in the performance of the heartbeat-counting task from the yoga naïve participants. Next, it was explored, if mean heart rate and HF-HRV activity [as measured in normalized units, HF-HRV (n.u.)] differed between the yoga practitioners and the yoga naïve participants during the exercise session, during the relaxation or during the affective judgment tasks. Comparisons between yoga practitioners and yoga naïve participants included non-parametric testing (Mann–Whitney-*U* tests, *Z* values). Finally, because the *n* = 5 participants who reported to have experience in yoga practice were assigned to both exercise groups, comparisons in HF-HRV (n.u.) activity between yoga practitioners and yoga naïve participants were also descriptively explored separately for the two exercise groups.

As shown in Table 2, descriptive statistics as well as statistical comparisons yielded no significant differences between yoga practitioners and yoga naïve participants in empathy, overall body awareness, performance in the heartbeat-counting task (pre- and post-exercise) or in mean heart rate or cardiac high frequency HF-HRV activity during the exercise session and during the affective judgment task (pre-exercise). Two outcome measures were significantly different between participants with self-reported yoga experience and those without self-reported yoga experience. First, yoga practitioners showed a higher mean score on the subscale “attention regulation” of the MAIA questionnaire (Mann–Whitney-*U*: *Z* = –2.03, *p* = 0.041). Second, participants with yoga experience showed a higher HF-HRV activity in the affective judgment task post-exercise (Mann–Whitney-*U*: *Z* = −2.11, *p* = 0.034) compared to those participants without yoga experience. Descriptive analyses suggest that higher HF-HRV activity in the affective judgment task (post-exercise) of the participants with yoga experience compared to those participants without yoga experience occurred in the exercise group 1, i.e., the group who exercised with body focus and breath instructions (see Table 2). Reanalysis of cardiac activity across experimental conditions in the two exercise groups without inclusion of the *n* = 5 participants with self-reported yoga experience did however not change the results reported in the Results section. Results of the re-analysis are summarized in Supplementary Table 2.

**TABLE 2:** Exploratory analysis: Descriptive statistics and comparisons between participants without self-reported yoga experience and participants with self-reported yoga experience.

		Participants without yoga experience (*n* = 25)		Participants with yoga experience (*n* = 5)	
	Measure	Score	SE	Score	SE
Empathy	Fantasy proneness	10.96	0.69	10.20	1.55
Empathy	Perspective taking	11.52	0.31	10.20	0.69
Empathy	Emotional concern	11.48	0.43	12.60	0.96
Empathy	Emotional distress	7.04	0.47	6.00	1.05
Empathy (IRI)	Empathy	33.96	1.01	33.00	2.27
MAIA	Noticing	11.82	0.45	10.10	1.01
MAIA	Not distracting	5.81	0.37	6.67	0.82
MAIA	Not worrying	5.63	0.38	6.60	0.84
MAIA	Attention regulation	17.85*	0.73	21.54*	1.62
MAIA	Emotional awareness	16.16	0.57	15.16	1.27
MAIA	Self regulation	10.16	0.48	11.35	1.07
MAIA	Body listening	5.76	0.52	6.33	1.15
MAIA	Trusting	7.87	0.41	7.73	0.91
HF-HRV (n.u.)	Affective task 1	40.01	3.07	48.08	6.85
HF-HRV (n.u.)	Relaxation	48.56	4.82	29.08	10.77
HF-HRV (n.u.)	Exercise session	26.03	1.76	27.25	3.94
HF-HRV (n.u.)	Affective task 2	36.71*	3.22	57.46*	7.21
HF-HRV (n.u.)	Exercise group 1	26.36* (*n* = 13)	3.12	75.37 (*n* = 2)	7.97
HF-HRV (n.u.)	Exercise group 2	47.91 (*n* = 12)	3.25	45.52 (*n* = 3)	6.50
mean HR	Affective task 1	79.29	2.55	74.56	5.71
mean HR	Relaxation	73.13	2.78	66.24	6.21
mean HR	Exercise session	81.57	1.72	81.06	3.84
mean HR	Affective task 2	74.02	2.22	71.23	4.97
Performance	Heartbeat counting 1	0.58	0.03	0.60	0.07
Performance	Heartbeat counting 2	0.65	0.04	0.68	0.09

SE: standard error, * indicates significant differences between the two groups (Mann–Whitney-U, Z-scores, see text for explanation).

## Discussion

This study had three major aims. First, investigate changes in heart rate variability (HRV) during a single session of yoga exercise and determine its changes during emotion processing before and immediately after the yoga exercise. Second, explore the importance of breathing and mindful body awareness instructions for (a) changes in HRV during the exercise and for (b) possible changes in body awareness and self-other referential emotion processing post-exercise. Third, investigate changes in self-other referential emotion processing immediately after the exercise by means of an experimental task rather than by self-report measures only. Data were recorded in a controlled laboratory setting in a mental and physically healthy all-female sample (final sample: *N* = 30), all university students, all relatively naïve about previous yoga experience. Participants were randomly assigned to two experimental exercise groups, half of the participants exercised the postures without controlled breathing and body focus, whereas the other half of the participants exercised the same postures with controlled breathing and mindful body awareness instructions (see Supplementary Table 1).

### Heart Rate Variability During the Exercise Session

Exercising for 30 min either with or without breath- and body awareness instructions changed cardiac activity significantly, both, when compared during exercise relative to before the exercise (during the affective task) as well as when cardiac activity was compared during the affective task pre- to post-exercise. Mean HR was equally pronounced during the affective task (pre-exercise) and during the exercise session. RMSSD as a measure of cardiac beat-to-beat variabilty significantly increased in both exercise groups during the exercise session in comparison to the affective task pre-exercise. HF-HRV (n.u.) significantly decreased and LF-HRV (n.u.) significantly increased during the exercise in comparison to the affective task pre-exercise. In summary, this differential behavior of cardiac activity to different mental and physical affordances of the task conditions (i.e., performing a cognitive-affective judgment tasks vs. 30 min of physical exercise) may well reflect healthy cardiac adaptability to different stressors, i.e., mental vs. physical. This supports previous research suggesting that cardiac activity, mental processing and bodily signal processing are intertwined and that these processes are reciprocally related at different functional processing levels (Porges, 2009).

Previous yoga studies focused on pre- to post-yoga HRV comparisons (Tyagi and Cohen, 2016). The results of these studies suggest a change in HRV before to after weekly yoga practice (Ross and Thomas, 2010; Zou et al., 2018; De et al., 2019). Only a few studies examined changes in HRV during a single yoga session. Most of these studies used a mix of yoga exercises without systematically comparing the effects of the conditions of exercising in combination with breath and mindfulness instructions vs. “posture alone” as in the current study (Telles et al., 2011; Kuppusamy et al., 2020; Shinba et al., 2020). Analysis of the data of the all-female sample in the present study did not support any differences in HRV measures between the two exercise groups during exercise. In other words, there was no indication that during the exercise session, practicing with controlled breathing and mindful body awareness instructions changed HRV measures differently from practicing the same exercises without controlled breathing and mindful body awareness instructions. Neither of the analyzed frequency- or time-domain HRV measures including HF-HRV (n.u.), LF-HRV (n.u.), RMSSD, or pNN50 differed on average significantly between the two exercise groups during the 30 min exercise session (see Figure 3). Also, mean heart rate (mean HR) did not differ significantly between the two exercise groups during the exercise session. The two exercise groups also did not differ in these measures from each other during the 3 min relaxation baseline preceding the exercise session. On the one hand, this suggests that exercising yoga postures with breath control and internal body focus is physically not more demanding than exercising yoga without such cognitively guided breath control and bodily self-control. On the other hand, this shows, that instructions asking for paying attention to one’s breath during the exercise and additionally being mindful by drawing attention toward felt-bodily sensations during the exercises have no superior effects on cardiac, particularly parasympathetic vagal activity during the exercise over practicing the same exercise without such explicit breath and internal body focus instructions.

One explanation of these no-group differences in HRV parameter modulation is that in the exercise group 1, breathing was not perfectly aligned with movements and that breath control during exercising was not at resonance frequency of 0.1 Hz. As far as breathing is concerned, the previous literature suggest that breathing interventions or yoga breathing training or biofeedback training that slow breathing to six cycles per minute (corresponding to a frequency of 0.1 Hz) can change cardiac activity significantly (Lehrer et al., 2000; Zaccaro et al., 2018; Kuppusamy et al., 2020). Moreover, when breathing and movements are temporally aligned such as during paced breathing and alternating muscle contraction exercise, significant changes in the high frequency measures of HRV have been observed (Chin and Kales, 2019). In the present study, the participants of the exercise group 1 were instructed to attend to their breath and perform body postures together with breathing. However, the participants were not instructed to paced breathing at six cycles or to breath at resonance frequencies of 0.1 Hz. The missing of resonance breathing is also seen in the measure of respiratory sinus arrhythmia (logRSA) suggesting no differences in respiration between the two exercise groups. The extent to which breath and movements were performed in temporal alignment by the participants of the present study cannot be answered. One possibility would have been to use self-report measures asking participants after each posture how much they felt breath and movement to be aligned to each other. In the present study, including such self-report questions in both exercise groups might have implicitly biased the participants (also those of the exercise group 2) to attend to their breath and take an internal body focus. Inclusion of self-reports at the end of the exercise session might have the disadvantage of producing hindsight and memory biases. Thus, answering this question would have required the recording of both, respiration and movement parameters in addition to cardiac activity.

The yoga postures comprised *asanas* (calm movements) that can be practiced without much physical effort and without heavy changes in breathing. At rest, with normal respiration frequencies, in healthy subjects, heart rate (HR) fluctuates with respiration and changes in the HF-HRV component are mainly triggered by changes in vagal peripheral nervous system (PNS) activity. As mentioned, specific slow-breathing yoga techniques but also heavy exercise can shift PNS to the range of the low-frequency LF-HRV component. In the current study, the yoga sessions elicited changes in HF-HRV (n.u.) and in LF-HRV (n.u.) when compared to the affective task or the relaxation period preceding the exercise session. The two exercise groups did not differ significantly in these HRV measures during the exercise session. Respiration was not directly controlled in the study, leaving open whether the two groups differed in frequency, depth or intensity of breathing during the exercise. As mentioned, estimation of respiratory sinus arrhythmia (logRSA) as normalized fluctuations of respiration-related beat-to-beat fluctuations showed no difference between the exercises groups during the exercise session. This suggests that breathing patterns during exercise might not have significantly differed between the two exercise groups and that exercising with and without breath and mindfulness body instructions might have been similarly demanding in both exercise groups. Nevertheless, validation of these observations and assumptions in future studies controlling respiration directly is warranted. Likewise, as already mentioned above, validation of the extent to which breathing was aligned with movements either spontaneously during the exercise (e.g., exercise group 2) or by explicit instructions (exercise group 1) requires investigation in further studies in which respiration and movement are controlled systematically and breathing and movement indicators are recorded and analyzed together.

### Heart Rate Variability, Self-Other Referential Emotion Processing, and Body Awareness Before and Immediately After the Exercise

Heart rate variability modulation during the affective judgment task following the exercise session differed significantly from HRV modulation during exercise. Moreover, HVR measures during the affective judgment task following the exercise session differed significantly from the HRV measures obtained during the affective judgment task before the exercise session. The results suggests a significant influence of the exercise on the modulation of cardiac activity during affective processing. Changes in HRV during the affective judgment task pre- to post exercise irrespective of the exercise group were found for the HRV measure, RMSDD showing significantly greater variability during the affective task post- compared to pre-exercise. This supports better short-term adaptability of cardiac activity during affective processing after a single bout of yoga exercise, whether practiced with or without breath control and body awareness.

Better short-term adaptability to an affective task pre-to post exercise as reflected in HRV measures such as RMSSD might result from cardiac heart rate recovery. Mean heart rate during the affective judgment task was significantly lower after exercise than mean HR during exercise and lower than mean HR during the affective judgment task before exercise. Previous research suggests that heart rate recovery from exercise is triggered by a release of parasympathetic cardiac control (Perini and Veicsteinas, 2003; Billman et al., 2015). The return of PNS activity after exercise occurs independently from sympathetic nervous system activity, which may remain elevated. In the present study, the relative decrease in mean HR after the exercise compared to during the exercise was significantly more pronounced in the exercise group 2, who had exercised without breathing and without body awareness instructions. This group additionally showed an increase in the high frequency HF-HRV (n.u.) component during the affective judgment task after exercise. The HF-HRV (n.u.) component during the affective judgment task was significantly higher in this group after exercise as compared to during the exercise as was the decrease in the LF-HRV (n.u.) component from (see Figure 4). The change in the pNN50 score during the affective task pre-to post exercise supported these group effects. The pNN50 scores were significantly more pronounced during the affective task after compared to before the exercise and again this was significantly more pronounced in the exercise group 2.

Overall, this suggests that a single session of yoga exercise of 30 min duration can improve cardiac affective regulation immediately after the exercise session. Moreover, this improved cardiac regulation after the exercise is characterized by an increase in parasympathetic cardiac control that is observed while performing an affective judgment task with self- and other-referential emotional stimuli. Furthermore, parasympathetic cardiac control during the affective judgment task is even more pronounced when the preceding exercise was practiced without breath and mindful body awareness instructions.

Yoga practice has been hypothesized to be beneficial for mental health in general and in particular for subjective emotion and mood regulation and for enhancing social cognition (Narasimhan et al., 2011; Tiwari, 2016a,b; Fiori et al., 2017). However, up to now, still missing in the literature are well-controlled experimental studies investigating the effects of exercise and of yoga in particular on emotion processing in relation to self-other referential processing in a pre to post design (Froeliger et al., 2012; Tiwari, 2016a,b). In a recent study, it was found that yoga practice increases a self-centered perspective during emotion processing. In turn, this seemed to facilitate attention to one’s own emotions while at the same time enhancing feelings of compassion and social connectedness (Fiori et al., 2017) but not necessarily feelings of altruism. In the present study, the experimental task chosen to investigate emotion processing pre to post exercise was an affective judgment task. Participants had to rate positive, negative and neutral words for emotional valence (positive, negative, or neutral) based on their gut feelings. In addition, words could vary in their self-other reference relating the content of the words either to the judger’s self or to a third person (e.g., “my fear” vs. “his fear”). Participants were faster and more accurate in their judgments of self-referential positive words. This self-positivity bias was also observed after the exercise.

The results of the present study are well in line with the observation that yoga facilitates a self-centered perspective that promotes self-referential processing (Fiori et al., 2017). However, the present results do not support the view that a single session of yoga exercise facilitates feelings of compassion (Kishida et al., 2018) or facilitates more empathetic judgments of other-referential stimuli. In contrast, the results suggest that a single session of yoga exercise leads to more adaptive emotion processing in general. In line with this assumption, participants responded faster to positive and negative words than to neutral words, irrespective of their self- or other-reference after the exercise session in comparison to before the exercise session. In addition, participants were more accurate in their affective judgments of negative, positive and neutral words, irrespective of their self- or other-reference, suggesting better emotion discrimination after the exercise session in comparison to before the exercise session. Moreover, participants were more accurate in the heartbeat-counting task after compared to before the yoga session and effects were found in both exercise groups. Taken together, this suggests that a single session of yoga exercise improves emotion discrimination and broadens the attentional focus to emotional stimuli as for instance suggested in the broaden-and-build model of emotion (Fredrickson and Branigan, 2005), while also generally improving appraisal of cardiac bodily signals. The correlational analyses showed that changes in HRV during the exercise and during the affective judgment task after the exercise as well as changes in reaction time and in accuracy measures of the affective judgment after exercise were overall not significantly correlated with the questionnaire measures of empathy or trait-like differences in body awareness.

Theoretical models of neurovisceral integration suggest a link between cardiac bottom-up and cerebral top-down controlled processing (Park and Thayer, 2014; Grol and De Raedt, 2020). Viewed from the perspective of the neurovisceral integration model, any intervention that triggers a coupling between heart, brain and mind should lead to neural activity changes in tightly intertwined brain networks thereby facilitating both, cognitively more flexible processing and physically better adaptability to certain tasks and stressors including emotional stimuli. In line with this assumption, 30 min of yoga exercise with or without breath- and body awareness instructions was accompanied by changes in HRV and in emotion processing pre- to post exercise. Of course, an interpretation of the current findings as support of the neurovisceral integration hypothesis needs validation by future studies investigating bodily changes in tandem with changes in brain activity.

Exercising for 30 min improved performance in the heartbeat-counting task. Although performance in the heartbeat-counting task has been linked to stable trait-like differences in the accuracy with which individuals can assess bodily signals such as one’s heartbeats (Garfinkel et al., 2015), recent research found state-like differences as well. Using variants of the heartbeat-counting task, it has been shown that performance in the task can be changed by experimental manipulation of attention to the Self and to the body (e.g., by exposing subjects to self-face photographs, self-relevant words or by means of mirror self-observation), which is in support of short-term (state-like) effects (e.g., Ainley et al., 2012, 2013). Interestingly, in the present study, instructions to focus on breathing and keeping a mindful body focus during the exercise seemed not to improve performance in the heartbeat-counting task more than exercising without such instructions. This supports results from the exercise literature that an internal attention focus that guides attention to body movements and internal bodily processes associated with the exercise is not always beneficial for mental and physical performance compared to exercising without an instructed attentional focus, especially in novices (Brick et al., 2014). Previous studies reporting positive effects of yoga on body awareness have compared groups of yoga professionals and regular yoga practitioners against yoga naive control groups and mainly used self-report psychological scales and questionnaires (Mantovani et al., 2016; Gibson, 2019).

Exploratory analysis comparing participants with and without self-reported yoga experience showed no difference between these groups in nearly most outcome measures. However, yoga practitioners scored higher on self-reported attention regulation of bodily signals (subscale of the MAIA questionnaire). This suggests a better ability of paying attention and redirecting attention to the body even when distracted in participants with yoga experience compared to participants without yoga experience (Mehling et al., 2012). This supports previous findings (e.g., Daubenmier et al., 2012) that yoga practitioners like meditation practitioners score higher in some measures of body awareness assessed with the MAIA questionnaire compared to non-practitioners. Moreover, exploratory descriptive analyses in the present study suggested that participants with yoga experience scored higher in HF-HRV activity in the affective judgment task (post-exercise) compared to those participants without yoga experience, especially when practicing with breath and mindfulness instructions (exercise group 1). Therefore, it is likely that individuals with expertise in yoga exercise, due to their previous experience, would have benefitted more from exercising with breath- and mindfulness guided instructions than exercising without such instructions (see Table 2), especially in cardiac adaptability to affective-cognitive processing. Similarly, it is likely that for yoga naïve individuals to improve cardiac and affective-cognitive processing by means of breath and mindfulness yoga exercise and to benefit more from it requires more than one session of exercise. Of note, due to the exploratory analysis and only the few participants with yoga experience, these assumptions are interesting but highly speculative. Nevertheless, exploratory analyses can guide hypothesis for future research investigating participants with and without yoga experience and using a similar exercise group design and multi-method approach as the present study.

## Limitations and Outlook

As already discussed above, the study has limitations. One limitation is the small sample size of an *N* = 30 all-female sample, all healthy, all university students having the same academic, educational background. There is evidence that in women, HRV parameters and heart rate (measured at rest) are modulated by the menstrual cycle (Sims et al., 2021). In a recent study, ANS activity (blood pressure, heart rate) and psychological well-being (self-report measures, e.g., depression, anger) were compared in female yoga practitioners (*n* = 25) as well as in controls (*n* = 25) across the menstrual cycle to examine whether yoga could influence pre-menstrual stress differently. The study found a main effect of menstrual cycle on ANS activity and partly on self-report measures of well-being. In addition, both groups differed in some of the ANS and psychological outcome measures (Kanojia et al., 2013). Although the study could only speculate about the potential mechanisms underlying these group-related differences and although the present study did not aim at exploring menstrual-cycle effects or its interaction with yoga or emotion processing, it would be interesting to investigate this question further in future studies.

In addition, the present results can and should not be generalized to different gender, age groups or individuals with a different yoga experience or to people diagnosed with health problems. As mentioned in the section “Discussion”, as far as yoga experience is concerned, *n* = 5 participants of the present sample who were equally distributed across the two exercise groups reported to have some experience with yoga in the past and the exploratory analysis comparing these participant groups is therefore tentative. An advantage of choosing a rather homogenous experimental sample with respect to sociodemographic background, mental and physical health, and previous yoga history, however, is that variability in these variables is controlled. Moreover, any bias in sampling or selection of participants was reduced by the study protocol including random selection of participants to the two study exercise groups. Moreover, university students constitute a high-educated, young and healthy population sample. Nevertheless, recent surveys have uncovered serious trends according to which about a quarter of all university students irrespective of country of residence report serious mental health problems being unable to cope with emotional and cognitive stress and academic demands. These problems seem to have nearly doubled during the current Covid-19 pandemic and have been reported to be more pronounced in female than male students (Gefen and Fish, 2012; Pelch, 2018). Health intervention initiatives comprising different exercises or single bouts of yoga exercise as the ones chosen in the present study might therefore constitute an important short-term intervention for buffering negative effects of chronic stress among university students. Of course, future studies should investigate mixed gender samples and additionally include control groups to validate the present findings. The current findings suggest immediate adaptive changes in HRV indices, self-other referential emotion processing and appraisal of body signals during and after 30 min of yoga exercise. Inclusion of further exercise or control groups in future studies could help compare the current findings to effects resulting from other types of exercises or non-exercise control interventions (Ross and Thomas, 2010). Moreover, as far as the role of breath and its alignment with movements during yoga exercise is concerned, future studies might benefit from movement control and especially from direct control of respiration such as respiration frequency, respiration depth and intensity instead of relying on respiration estimates such as in the present study.

## Conclusion

Various beneficial health effects have been attributed to regular yoga practice. Although the effects on mental health, physical health and well-being attributed to regular yoga practice are diverse, recent meta-analytic research suggested that not all of these effects have proven so far in experimentally well-controlled settings (Büssing et al., 2012). Especially, there is lack of experimental studies investigating immediate effects of yoga exercise on cardiac adaptability and this in relation with emotion and self-referential processing including self-other processing (Park et al., 2020). Therefore, despite restrictions in the generalizability of the results of the present study, the results of the present study add to a yet underexplored field of research. Given the very few previous yoga studies with an experimental design that have been examining immediate effects of yoga on a set of physiological and psychological health parameters and indicators, the present study can be considered relevant. Comparing cardiac activity (HRV), (a) across experimental conditions, and (b) investigating self-other referential emotion processing in a pre- to post design after a single session of yoga exercise, while (c) additionally exploring the role of yoga-related breath and mindfulness instructions, and (d) changes in body awareness, revealed a number of interesting findings. These were left open in previous studies and should be examined further in future studies.

## Data Availability Statement

The original contributions presented in the study are included in the article/Supplementary Material, further inquiries can be directed to the corresponding author/s.

## Ethics Statement

Participation in the study was voluntary, participants were instructed in detail that they could reject from participation throughout the study without negative consequences. Participants had to give written informed consent to confirm the exclusion criteria and were debriefed about the purpose of the study, following ethical guidelines and study protocol standards, used in the department’s previous physical activity studies, which had been approved by the local ethics committee of Ulm University (https://www.uni-ulm.de/einrichtungen/ethikkommission-der-universitaet-ulm/). Therefore, for this study, no ethics approval was submitted before the start of the study.

## Author Contributions

CH: conception and design of the study; drafting, writing, and revision of the manuscript; data analysis, validation and interpretation; conception and programming of the HisMine paradigm; creation and design of tables and figures; and final approval of the manuscript version to be published.

## Conflict of Interest

The author declares that the research was conducted in the absence of any commercial or financial relationships that could be construed as a potential conflict of interest.

## Publisher’s Note

All claims expressed in this article are solely those of the authors and do not necessarily represent those of their affiliated organizations, or those of the publisher, the editors and the reviewers. Any product that may be evaluated in this article, or claim that may be made by its manufacturer, is not guaranteed or endorsed by the publisher.

## Footnotes

1
https://www.polar.com/en
