# Supplementary material for: Can Yoga Boost Access to the Bodily and Emotional Self? Changes in Heart Rate Variability and in Affective Evaluation Before, During and After a Single Session of Yoga Exercise With and Without Instructions of Controlled Breathing and Mindful Body Awareness in Young Healthy Women
Source: Front Psychol. 2021 Dec 3;12:731645. doi: 10.3389/fpsyg.2021.731645 (PMC8678535; doi:10.3389/fpsyg.2021.731645)
Supplement: Supplementary file 1 [file Table_1.pdf]

Herbert, C.: Yoga, HRV, and Emotional Processing

https://doi.org/10.3389/fpsyg.2021.731645

Supplementary Material

Herbert, C. (2021)

Can Yoga Boost Access to the Bodily and Emotional Self? Changes in Heart Rate Variability and in Affective Evaluation Before, During and After a Single Session of Yoga Exercise With and Without Instructions of Controlled Breathing and Mindful Body Awareness in Young Healthy Women

Supplementary Material Table 1: Overview of the exercises performed in the exercise group 1 and the exercise group 2, respectively.

Yoga Group 1
yoga postures with instructions of voluntary breath control and mindful body awareness

Yoga Group 2
yoga postures without instructions of breath control and mindful body awareness

**Exercises**<br><br>**guided by instruction**		**Exercises**<br><br>**guided by instruction**
Relaxation 3 minutes	**Shavasana (dead body pose)**	Relaxation 3 minutes
**Posture 1**<br>lift left leg perpendicular to the ceiling, right leg remains loosely on the floor, repeat with legs turned<br><br>instruction breath control:<br>inhale: lift leg, exhale: bring leg down, repeat for five breaths at own pace, relax<br><br>instruction mindful body awareness:<br>attention to legs, feel into body, feel change between left and right leg	**Asana leg stretching Exercise 1**<br><br>**3 min**	**Posture 1**<br>lift left leg perpendicular to the ceiling, right leg remains loosely on the floor, hold for 20 seconds (20, 19, 18 ...), pause for 10 seconds, right leg, (20, 19, 18 ...)<br><br>no breath instruction<br>no body focus instruction
**Posture 2**<br>left foot on the ground, left knee to chest, grasp it with both hands, repeat with right leg during next inhalation<br><br>instruction breath control:<br>inhale when knee to chest, breathe into posture<br><br>instruction mindful body awareness:<br>feel into body, feel change between left and right leg	**Asana psoas stretch Exercise 2**<br><br>**3 min**	**Posture 2**<br>lift left leg to chest and grasp with both hands, stay in this position for 30 seconds (30, 29, 28 ...), stretch for 30 seconds (30, 29, 28 …)<br><br>no breath instruction<br>no body focus instruction

https://doi.org/10.3389/fpsyg.2021.731645

**Posture 3**<br>both feet on floor, legs bent, arms loose next to body, extend arms to ceiling, bring arms behind the head while inhaling, bring arms back to floor while exhaling, stretch, repeat 10 times, breathe in own pace<br><br>instruction breath control:<br>inhale-exhale in sync with movement, breathe into posture<br><br>instruction mindful body awareness:<br>feel into body, feel change	**Asana**<br>**shoulder bridge**<br>**Exercise 3**<br><br>**3 min**	**Posture 3**<br>both feet on floor, legs bent, arms loose next to body, on command raise hands, repeat 10 times<br><br>no breath instruction<br>no body focus instruction

**Posture 4**<br>both feet on floor, arms loose next to body, inhale, pull your navel towards the ceiling, form a hollow back, exhale, press back against floor, repeat exercise for 10 breath at own pace, breathe in and out vigorously, with the last inhalation, lower your legs and place them loosely on the floor<br><br>instruction breath control:<br>inhale-exhale in alignment with the movement.<br><br>instruction mindful body awareness:<br>feel how pelvis moves, feel how body goes with the movement, feel the stretch in your back as you inhale.	**Asana**<br>**pelvic swing**<br>**Exercise 4**<br><br>**3 min**	**Posture 4**<br>both legs on the floor, put arms as close as possible to body, lift buttock off the floor, the back, legs and floor form a triangle, hold for 40 seconds, when time is up take a 20-second break<br><br>no breath instruction<br>no body focus instruction
**Posture 5**<br>take both knees, bring them to the chest, embrace knees with both arms, pull legs close to the body, begin to breathe deeply into this position, keep breathing in your own rhythm, bring legs back to the floor, place arms loosely next to the body, relax while lying down, stretch out again with pleasure, get into seat<br><br>instruction breath control:<br>breathe into your stomach, breathe deeply into this posture and then release<br><br>instruction mindful body awareness:<br>feel heaviness of legs, try to locate changes, feel your internal organs	**Asana**<br>**Apanasana**<br>**knee to chin**<br>**Exercise 5**<br><br>**2 min**	**Posture 5**<br>pull both legs to the chest, embrace with arms, lift head to your knees, hold for 40 seconds, bring legs and upper body back to the floor, 20 seconds break, repeat posture, keep your head at your knees, hold for 40 seconds, release posture, take a break<br><br>no breath instruction<br>no body focus instruction

**Posture 6**<br>extend legs forward, keep back straight, hold posture for three deep breaths, keep back as straight as possible, with the next inhalation stretch upper body and arms forward as far as possible, exhale and come back to starting position with back straight, inhale and stretch forward, repeat for seven breaths at own pace, stretch a little further with each inhalation, hold one more breath in the posture, now breathe out and relax, relax body, shake legs out.<br><br><u>instruction breath control:</u><br>align breath and movement<br><br><u>instruction mindful body awareness:</u><br>feel into body	**Asana**<br>**the stick plus bend forward**<br>**Exercise 6**<br><br>**3 min**	**Posture 6**<br>get into the seat, stretch legs, keep back straight, bend back forward, stretch arms to the feet, keep back straight, hold stretch for 30 seconds, release the posture, put back into starting position, stretch arms upwards, 10 second pause, repeat once more …<br><br><u>no breath instruction</u><br><u>no body focus instruction</u>
**Posture 7**<br>sit cross-legged, with next breath spread arms, stretch yourself up, put hands back on the floor while exhaling, inhale and stretch up, repeat for five breaths at own pace, stretch yourself far forward, let arms slide slowly back to the floor<br><br><u>instruction breath control:</u><br>align breath and movement<br><br><u>instruction mindful body awareness:</u><br>feel into the movement	**Asana**<br>**Sukhasana and counter-stretch**<br>**Exercise 7**<br><br>**2 min**	**Posture 7**<br>sit cross-legged, back remains straight, lift left arm up, bring hand behind head to the right shoulder, bring right hand to the elbow of your left arm and pull it to the right, hold position for 30 seconds, put arm back down, shake it out vigorously, repeat exercise with right arm<br><br><u>no breath instruction</u><br><u>no body focus instruction</u>

**Posture 8**<br>stand with both feet and feel the ground, breathe in and move arms upward past the body, next stretch yourself, breathe out vigorously and let arms fall down; repeat five times with five breaths at your own pace, exhale, bring arms and upper body down with back straight, let arms dangle downward, bring upper body back up, vertebra by vertebra, stretch arms over the body, exhale and come back down, repeat five times with breaths at own pace, last inhalation, stretch yourself up, exhale and let arms fall back down<br><br><u>instruction breath control:</u><br>align breath and movement<br><br><u>instruction mindful body awareness:</u><br>open eyes and feel into the movement	**Asana**<br>**Uttanasana stretching and bending forward while standing**<br>**Exercise 8**<br><br>**3 min**	**Posture 8**<br>stand with both feet, stretch arms up and upper body backwards, stretch up as far as possible, let arms and upper body fall down, get hands to feet, hold position for 30 seconds, come back up, stretch arms up again and stretch back a little, bring upper body back down and hold the stretch for 30 seconds again<br><br><u>no breath instruction</u><br><u>no body focus instruction</u>

https://doi.org/10.3389/fpsyg.2021.731645

**Posture 9**<br>get into four-legged position, knees, hands parallel to shoulders and hips, inhale, hollow back, push back down, hold head up, exhale while straightening the back, pull head to chest, repeat this exercise for ten time, breathe in own rhythm, stretch a little more with each exhale, enjoy stretching of spine, exhale and stretch buttock back, rest upper body thighs<br><br><u>instruction breath control:</u><br>align breath and movement 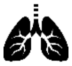<br><br><u>instruction mindful body awareness:</u><br>feel and enjoy the movement	**Asana**<br>**the cat**<br>**Exercise 9**<br><br>**2 min**	**Posture 9**<br>get into four-legged position, knees, hands parallel to shoulders and hips, straighten back and hunch back, pull head to chest, hold posture for 30 seconds, push back down and form a wooden cross, hold posture for 30 seconds<br><br><u>no breath instruction</u><br><u>no body focus instruction</u>
**Posture 10**<br>put forehead on the floor, stretch hands back, place hands next to the body, stay in this position and breathe deeply and evenly<br><br><u>instruction breath control:</u><br>align breath and movement 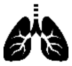<br><br><u>instruction mindful body awareness:</u><br>feel into the movement	**Asana**<br>**the child**<br>**Exercise 10**<br><br>**2 min**	**Posture 10**<br>put upper body and leg on the floor, stretch in all directions for 30 seconds<br><br><u>no breath instruction</u><br><u>no body focus instruction</u>
