# Supplementary material for: Can Yoga Boost Access to the Bodily and Emotional Self? Changes in Heart Rate Variability and in Affective Evaluation Before, During and After a Single Session of Yoga Exercise With and Without Instructions of Controlled Breathing and Mindful Body Awareness in Young Healthy Women
Source: Front Psychol. 2021 Dec 3;12:731645. doi: 10.3389/fpsyg.2021.731645 (PMC8678535; doi:10.3389/fpsyg.2021.731645)
Supplement: Supplementary file 2 [file Data_Sheet_1.pdf]

Herbert, C.: Yoga, HRV, and Emotional Processing

https://doi.org/10.3389/fpsyg.2021.731645

Supplementary Material

Herbert, C. (2021)

Can Yoga Boost Access to the Bodily and Emotional Self? Changes in Heart Rate Variability and in Affective Evaluation Before, During and After a Single Session of Yoga Exercise With and Without Instructions of Controlled Breathing and Mindful Body Awareness in Young Healthy Women

Results – Exploratory Analysis

All-female sample (n=5 participants reporting yoga experience excluded)

Cardiac activity and heart rate variability

Analysis of cardiac parameters without the data of the five participants who reported to have practiced yoga in the past yielded the same effects as the analysis reported in the manuscript in section 4.1. The analysis of changes in mean heart rate (mean HR) as well as of changes in the frequency-domain HRV parameters (HF-HRV (n.u.) (and also LF-HRV (n.u.) and pNN50) as a function of the experimental conditions and two exercise groups, showed significant main effects of the within-subject factor "condition" and significant interactions of the factors "condition" x "group"; see Table 4. RMSSD showed a main effect of "condition" as reported in 4.1

Supplementary Table 2

Outcome Measure	Main effect condition	Interaction effects condition x group
HF-HRV (n.u)	$F(2,46)=19.54$ $p<.001$	$F(2,46)=9.82$ $p<.005$
LF-HRV (n.u)	$F(2,46)=19.54$ $p<.001$	$F(2,46)=9.82$ $p<.005$
RMSDD	$F(2,46)=8.56$ $p=.006$	$F(2,46)=4.98$ n.s.
pNN50	$F(1,23)=4.24$	$F(1,23)=14.97$

	p=.05	p<.001
mean HR	*F*(2,46)=16.214<br>p<.001	*F*(2,46)=4.316<br>p=.019

Similarly, post-hoc comparisons of the significant interaction effects comparing the two exercise groups against each other yielded the same effects as the analysis reported in the manuscript in section 4.1. The analysis that included only yoga naïve participants in both exercise groups showed that the relative decrease in mean HR (from during the exercise to during the affective task post-exercise) was significant only in the participants of the exercise group 2 (who exercised without instructions of breath control and without instructions of mindful body awareness), (*F*(1,23)=56.53, p<.001). In addition, planned contrast comparisons including only yoga naïve participants in both exercise groups showed that the increase in HF-HRV (n.u.) from during the exercise to during the affective task post-exercise was significant in the exercise group 2 only (*F*(1,23)=40.21, p<.001). The HF-HRV (n.u.) component did not differ between the two exercise groups during the exercise session nor during the affective task pre-exercise (all p>.1). The low frequency LF-HRV (n.u.) component showed the reverse effects, and pNN50 was significantly higher in the affective task after the exercise in the exercise group 2 compared to the exercise group 1 (*F*(1,23)=4.30, p=.049), whereas the two exercise groups did not differ in this measure during the affective task before exercise (p>.1).
